# Plastic Solid Waste (PSW) in the Context of Life Cycle Assessment (LCA) and Sustainable Management

**DOI:** 10.1007/s00267-019-01178-3

**Published:** 2019-06-22

**Authors:** Ana Antelava, Spyridon Damilos, Sanaa Hafeez, George Manos, Sultan M. Al-Salem, Brajendra K. Sharma, Kirtika Kohli, Achilleas Constantinou

**Affiliations:** 1 Division of Chemical & Petroleum Engineering, School of Engineering, London South Bank University, London, SE1 0AA UK; 2 Department of Chemical Engineering, University College London (UCL), London, WCIE 7JE UK; 3 Environment & Life Sciences Research Centre, Kuwait Institute for Scientific Research, P.O. Box 24885, Safat, 13109 Kuwait; 4 Illinois Sustainable Technology Center, Prairie Research Institute, University of Illinois, Urbana-Champaign, 1 Hazelwood Dr., Champaign, IL 61820 USA

**Keywords:** Pyrolysis, Plastics, Sustainable Management, Recycling, Energy, LCA

## Abstract

Over the past few decades, life cycle assessment (LCA) has been established as a critical tool for the evaluation of the environmental burdens of chemical processes and materials cycles. The increasing amount of plastic solid waste (PSW) in landfills has raised serious concern worldwide for the most effective treatment. Thermochemical post-treatment processes, such as pyrolysis, seem to be the most appropriate method to treat this type of waste in an effective manner. This is because such processes lead to the production of useful chemicals, or hydrocarbon oil of high calorific value (i.e. bio-oil in the case of pyrolysis). LCA appears to be the most appropriate tool for the process design from an environmental context. However, addressed limitations including initial assumptions, functional unit and system boundaries, as well as lack of regional database and exclusion of socio-economic aspects, may hinder the final decision. This review aims to address the benefits of pyrolysis as a method for PSW treatment and raise the limitations and gaps of conducted research via an environmental standpoint.

## Introduction

Production of plastics has increased drastically over the past century, from a mere 1.3 million tonnes in 1950 to >322 million tonnes in 2015 (PE 2016). A global increase of plastics consumption is also noted with a rate of 4% per annum (Miandad et al. 2016). The associated cost of managing plastic solid waste (PSW) drives several countries and communities alike to discard it in open landfill sites. This leads to the accumulation of plastic commodities and articles as a major component in the solid waste (SW) stream. PSW is bulkier than other organic refuse, thus occupies larger space in landfills. Various advances occurred within the past three decades in SW recycling and valorisation. Regardless, ~9.5% of the total plastic produced over the period from 1950–2015 has been recycled, while 12.5% has been incinerated and 78% is still discarded in landfills (Geyer et al. 2017).

PSW can be categorised depending on its source or point of origin, i.e. municipal, industrial, medical, etc. However, the majority of PSW is generated from households and commercial sources, which combined, are referred to as municipal plastic waste (MPW). This type of SW mainly consists of the following plastic resin types: polyethylene (PE), polypropylene (PP), polystyrene (PS), polyethylene terephthalate (PET) and polyvinyl alcohol (PVC) (Miandad et al. 2017). MPW is typically thermoplastics, which are thermally recyclable due to their non-resistance to heat. According to the ISO 15270 (2008), PSW can be recycled and treated to produce raw materials, and the production of high calorific compounds can be used as fuels for energy production. MPW is treated by an ascending order of preference from reprocessing and extrusion, to recovering utilities and energy. For example, mechanical recycling results in plastic pelletization and subsequently raw plastic materials. On the other hand, chemical recycling processes lead to polymer cracking, to monomers allowing the production of polymers and fuels. The management of PSW in general will rid the environment of the accumulation of PSW, and prevent pollution problems from landfilling, such as toxins leaching that can contaminate ground water aquifers (Al-Salem et al. 2015).

Incinerating SW has become a popular choice of treatment as a waste-to-energy (WtE) management technology. Nevertheless, incineration of PSW is reported to cause air and groundwater pollution problems related to the plastic type and content in the waste, as well as the process conditions, due to the emissions of GHG, SOx, particles, volatile organic compounds (VOCs) and polycyclic aromatic hydrocarbons (PAHs) (Al-Salem et al. 2009). The European Union (EU) has established permissible emission limits and guidelines described in the Council Directive 2000/76/EC. It was also previously concluded that different thermoplastics result in varying levels of PAH post treatment via incineration (Li et al. 2001). Typically, PE and PP will result in high PAH levels measured in the flue gas of incineration units, although PVC will have higher levels of PAHs in the bottom ash recovered rather than the flue gas. This is attributed to the fact that PVC will decompose at higher temperatures at a stage where the additives to the resin will coagulate in the ash.

It is well noted at this stage of technical development, that environmental impacts of processes are divided into three main categories, namely as energy related, climate change related and eco-toxicological impacts (Lazarevic et al. 2010). In case of plastics with significant chlorine content, incineration causes the formation and emission of dioxins and furans, such as polychlorinated dibenzo para dioxins (PCDD) and polychlorinated dibenzo furans (PCDF) (Lazarevic et al. 2010). Process conditions are of major importance as incomplete combustion of the PSW could lead to the formation of carbon monoxide (CO) and smoke (Verma et al. 2016). In case of high nitrogen content plastics, such as polyurethanes (PU), incineration could lead to excess emissions of nitrogen oxide (NO) and nitrogen dioxide (NO_2_), with a dramatic increase of the global warming potential (GWP) (Al-Salem et al. 2009). Thus, various thermo-chemical treatment (TCT) methods such as hydrogenation, gasification and pyrolysis became important for the management of MPW (Nizami et al. 2015).

Pyrolysis presents several advantages for treating PSW, namely solid plastics originating from the municipal sector. Pyrolysis involves the degradation of the constituting polymers of the plastic materials by heating them in inert (non-reactive) atmospheres. The process is typically conducted at temperatures between 350–900 °C and produces carbonized solid char, condensable hydrocarbon oil and a high calorific value (CV) gas. The product’s selectivity and yields of product fractions depend on the plastic type along with process conditions (Al-Salem et al. 2017). It is divided into two main types, thermal (without the presence of catalysts) and catalytic pyrolysis. Thermal pyrolysis produces liquids with low octane value and higher residue contents at moderate temperatures (Seth and Sarkar 2004). The gaseous products obtained by thermal pyrolysis typically require upgrading to be used as a fuel (Panda et al. 2010). Pyrolysis can also be conducted catalytically; reducing the temperature and reaction time required for the process and allowing the production of hydrocarbons with a higher CV value such as fuel oil (Almeida and Marques 2016). The presence of catalysts in pyrolysis also aids the evolution of gasoline and diesel range products (Aguado et al. 2000) and gives an added value to pyrolysis. The cracking efficiency of these catalysts depends on their chemical and physical characteristics. These properties promote the breaking of carbon to carbon (C–C) bonds and determine the length of the chain of the obtained products.

One of the main aims of the EU environmental policies is to integrate the environmental sustainability with economic growth (Tarantini et al. 2009). There is an environmental concern about the increase in conventional PSW management by mechanical means, and whether it is the most sustainable practice. These concerns are due to high energy demands around various European communities. Decision makers need to evaluate technical, environmental and economic aspects of waste management techniques. Environmental impact assessment (EIA) and inventory analysis are prime examples of such techniques. However, life cycle assessments (LCA) can provide a more in-depth framework to evaluate the waste management strategies, identify environmental impacts and hot spots with respect to the waste treatment hierarchy. LCA evaluates environmental burdens and potential impacts associated with processes, by gathering an inventory of inputs and outputs and interpreting the results of the study.

To perform state-of-the-art LCA studies for PSW technologies, a systematic overview of assessment processes is required. The aim of this review is to provide such an overview based on the existing LCA studies of PSW processes reported in literature. In particular, a comprehensive review and analysis of the pyrolysis process is evaluated in context of its environmental performance through LCA. The associated benefits and burdens of this process are detailed and reported from an LCA standpoint. This was done to be able to compare various scenarios that have incorporated pyrolysis to valorise PSW. This work can also aid decision makers (and takers) in understanding the benefits associated with pyrolysis. Various research gaps are detailed and showcased for the reader’s consideration. To the best of the author’s knowledge, no such work has been attempted in the past.

## Plastic Waste Management Options and Processes

Table 1 provides a list of the major advantages and disadvantages for the main plastic waste management techniques. The major practicing routes for disposing waste plastics are; landfill, mechanical recycling, and energy recovery (Al-Salem et al. 2009; Lazarevic et al. 2010). Recycling and reuse are not suitable for all waste streams, thus a great amount of MSW ends up in landfills and waste-to-energy (WtE) plants (Margallo et al. 2018). Gu et al. (2017) has investigated the life cycle of mechanical plastic recycling in China. The results have shown that mechanical recycling is a superior alternative in most environmental aspects, compared with the production of the virgin plastics. Virgin composite production has an impact which is almost four times higher than that of the recycled composite production (Gu et al. 2017). Despite odorous emissions released during meltdown of waste plastics and soil contaminations, mechanical recycling is generally an environmental-friendly approach for waste plastic disposal.

**Table 1:** Advantages and disadvantages of plastic waste management techniques

Treatment processes	Advantages	Disadvantages/limitations
Landfill	• Relatively low cost and easy implementation (Cheng and Hu 2010).	• Does not achieve the objectives of reducing volume of MSW and converting MSW into reusable fuels (Cheng and Hu 2010). • Requires large area of land (Cheng and Hu 2010). • Persistent organic pollutants (POPs) can be released from landfilling (Melnyk et al. 2015). • Pollution and soil contamination may serve as breeding ground for pests and diseases (Cheng and Hu 2010).
Mechanical Recycling	• Reduction in Greenhouse Gases (GHG) (Makuta et al. 2000). • Reduction in Carbon footprint (Dormer et al. 2013). • Recycled plastics represent a save of 20–50% in terms of the market prices compared with virgin counterparts (Gu et al. 2017).	• Can only be performed (effectively) on single-polymer plastics, e.g. PE, PP, PS, etc. (Al-Salem et al. 2009). • Separation, washing and preparation of PSW are all essential to produce high quality end-products (Al-Salem et al. 2009).
Gasification	• Requires short residence time and has high conversion yields (Al-Salem et al. 2017). • Produces high CV gas with a completely combusted residual char (Borgianni et al. 2002). • Can either produce large amounts of char and ash or convert waste to small amounts of char and ash with large amounts of syngas (Begum et al. 2014).	• Requires high operational temperatures (Panepinto et al. 2015). • Careful feedstock preparation by crushing, shredding and sieving with controlled moisture content has to be achieved (Panepinto et al. 2015). • Produces large amounts of tars between 0.1 and 10% of the product gas. Compressed tar may cause serious problems to the process and equipment (Milne and Evans 1998). • Requires high operational costs due to feed pre-treatment, oxygen consumption as well as syngas cleaning costs (Panepinto et al. 2015).
Pyrolysis	• Pyrolysis produces a high CV fuel that could be easily marketed and used in gas engines to produce electricity and heat (Demirbas 2004). • No need of flue gas treatment (Al-Salem et al. 2009). • Does not require as many feedstock pre-treatments as other methods (Al-Salem 2017). • Does not form dioxins due to inert reaction atmosphere free from oxygen (Chen et al. 2014).	• Requires handling of produced char (Ciliz et al. 2004). • Requires treatment of the final fuel produced if specific products are desired (Al-Salem et al. 2009). • Insufficient understanding of the underlying reaction pathways, which has prevented a quantitative prediction of the full product distribution (Al-Salem et al. 2009).

Municipal solid waste incineration is another robust waste treatment method, which not only reduces waste volume but also allows for the efficient recovery of energy. However, it requires high construction, installation and maintenance costs (Margallo et al. 2018). Gasification process involves the heating of the feedstock materials under a controlled amount of oxygen to produce synthesis gas without fully oxidizing the feedstock to carbon dioxide. The synthesis gas can then be used to generate power or heat or be converted by catalytic Fisher-Tropsch synthesis to hydrocarbons (Benavides et al. 2017). Several LCA studies have compared the MSW treatment techniques such as landfill, combustion, gasification to pyrolysis. This analysis agrees that the pyrolysis technique offers more environmental benefits, such as reduction of GHG emissions and consumption of fossil fuels (Benavides et al. 2017).

Pyrolysis is a thermal decomposition process of organic materials in the absence of oxygen into char, oil and gas (Sheth and Babu 2009, Wang et al. 2015). An oxygen-free environment prevents the oxidation of the hydrocarbon which would have reduced the heating values of the product fuel. The proportion of the pyrolysis products such as liquid fuel, gas and char depends on the feedstock composition as well as the conditions of the process (Benavides et al. 2017). The produced liquid oil can have many applications, for example, it can be used as an energy source. Its potential use as a transport fuel source might require further upgrading and blending with diesel to improve its characteristics, as it contains a high number of aromatics. The use of pyrolysis oil, together with diesel as transport fuel, was successfully tested at different ratios in past research (Demirbas 2004; Gardy et al. 2014; Islam et al. 2010, Miandad et al. 2017). Another product of pyrolysis is char. Char produced from PS plastic wastes has a higher heating value (HHV) of 36.29 MJ/kg (Syamsiro et al. 2014), therefore it has the potential to be used as an energy source. Several researchers have activated pyrolysis char using steam (Lopez et al. 2011), hydrogen peroxide (Heras et al. 2014) or by thermal activation (Jindaporn and Lertsatitthanakorn 2014). Activation of char increases its surface area that improves the ability to adsorb the heavy metals, odours and toxic gases (Miandad et al. 2017).

Over the past year there have been several case studies on the life-cycle assessment of waste treatment (plastic, municipal, etc.), or biomass treatment via pyrolytic methods for energy or fuel production. Demetrious and Crossin (2019) have examined and compared the waste treatment via landfill, incineration and gasification-pyrolysis showing the importance of pyrolysis on reducing the greenhouse gas (GHG) potential, although landfill method requires less energy and is preferred to gasification-pyrolysis route. It is noteworthy that they provide an insightful discussion on the limitations of the LCA methodology of this study, which is related to the geographical scope, the electricity mix assumed, as well as the limitation of the LCA on the environmental impacts associated with plastic reaching out in the natural environment causing micro-plastic ingestion and marine entanglement. Finally, their study paved the way for policy amendment on the waste management.

Vienescu et al. (2018) studied the use of pyrolysis to produce synthetic fuels via an LCA approach. Despite the promising results, similar LCA studies must consider the wide range of environmental impacts that occur during the synthesis production. This is due to the rise of environmental burdens in comparison to the diesel and petrol production processes. Therefore, the materials used in system construction, as well as different allocation methods for stover and pyrolysis by-products, need to be investigated for their environmental and socioeconomic trade-offs. Barry et al. (2019) conducted an environmental and economical analysis on municipal sewage sludge via pyrolysis. Based on their findings, the two pyrolysis scenarios performed better than the incineration scenarios with respect to the impact categories of global warming potential, and freshwater ecotoxicity, with the use of the biochar as a coal substitute offering the greatest greenhouse gas reductions.

Khoo (2019) assessed PSW recovery into recycled materials, energy and fuels in Singapore through LCA. The waste treatment options included mechanical recycling, pyrolysis and gasification. The work highlights the normalisation and weighting factors on the LCA analysis in accordance to the relative importance of environmental impacts and sustainability indicators. Different normalization methods can be applied which will result in different outcomes, and weighting factors can also be influenced by altered political views or agendas, geographical settings, environmental regulations, or even cost. Therefore, LCA results are biased on the system boundaries and the weighting factors considered in the analysis.

Gear et al. (2018) developed a toolkit for process design via LCA, focusing on the thermal cracking process for mixed plastic waste. The case study focused on the products of recycling technologies process; however, the toolkit performs hotspot analysis and multivariable optimization that includes environmental performance across the entire range of possible weighting. Their result indicates the importance of integrating process optimization with environmental impact assessment via data analysis and LCA.

Several companies utilise different waste management technologies, in order to, convert PSW to fuel and other valuable products. Within the European Economic Area (EEA) agreement countries, there is a significant number of industrial partners that utilise thermal waste-to-fuel (WtF) technologies including Cynar plc, Plastoil, Promeco, Syngas Products Group, Plastic Energy, Recycling Technologies and Enval Ltd (Haig et al. 2017). Amongst these companies, Syngas Products Group Ltd focuses on non-recyclable waste feedstock to energy, while utilising a combined process of pyrolysis-gasification for the synthesis of renewable gas of high calorific value. The company’s plant in Canford, Dorset (UK) has a capacity of 10 ktpa of PSW feedstock input with a 0.8 MWe unit for power generation. The company also plans to expand and scale up the facility to 100 ktpa input and 8 MWe output (Syngas Products Group 2019). They established a fully commercial plastic liquefaction facility on the island of Hokkaido. Plastic Energy Co. has a patented thermal anaerobic conversion technology aimed at converting PSW into feedstock for plastics production or alternative low-carbon fuels. The company has two recycling plants in Seville and Almeria (Spain) which have been in operation since 2014 and 2017, respectively. For every tonne of end-of-life PSW processed, 850 litres of chemical pyrolysis oil (TACOIL) is produced. The company aims to process 200,000 tonnes of plastic by 2020 (Plastic Energy 2019).

Recycling Technologies have developed a process methodology for plastic recycling via converting the plastic waste to fuel and its capacity reaching up to 9000 tpa. They have also commercialised four special ultra-low sulfur oils (reaching less than 0.1% sulfur content) derived from recycled plastics—called Plaxx—which can be used as a fuel substitutes or feedstocks to produce plastics or wax (Recycling Technologies 2019). Enval ltd. focuses on microwave-induced pyrolysis to process plastic aluminium laminates. Recycling aluminium through the Enval process leads to energy savings of up to 75%. With a purity exceeding 98% and a minimum metal yield of 80%, it can be directly reintroduced to the resmelting process. A typical Enval plant produces 200–400 tonnes of aluminium a year. The generated pyrolytic oils can be used as chemical feedstock or for energy generation. The Enval process can be controlled to adjust yield of the gases and oils according to the operator’s requirements. Enval plants can operate at a feed rate of up to 350 kg per hour, which equates to a nominal capacity of 2000 tonnes per year (Enval 2019). Etia Ecotechnologies has developed an innovative patented pyrolysis process Biogreen® that is operating since 2003 (ETIA Group 2019).

In addition, a significant number of companies based in the United States of America (USA) perform pyrolysis to produce fuel from plastics, such as Agilyx (2019), Global Renewables and Vadxx, Climax Global Energy, Envion, Plastic Advanced Recycling Corp, Plastic2Oil and PolyFlow (Haig et al. 2017). Agilyx was founded in 2004 and is based in Oregon, USA. It has operated as a pyrolysis plant that processes rigid PSW to recycle plastics into low carbon synthetic crude oil, and in 2018 opened a polystyrene to styrene monomer facility (Butler et al. 2011). The Vadxx plant is utilising no-recyclable plastic to produce fuel via continuous pyrolytic process. The company has a plant in Ohio, USA of 25,000 tonnes plastic annual capacity, to produce solid (solid carbon-based fuel), liquid (naptha and diesel) and synthetic gas fuels (Bailey 2014). Biogreen® The Plastic2Oil Inc. has developed their own in-house technology that derives ultra-clean, ultra-low sulphur fuel that does not require further refining from waste plastic. The conversion ratio of the waste plastic into fuel is about 86% with 2–4% of the resulting product being Carbon Black. The company reports that the process’ emissions are lower than that of a natural gas furnace of the similar size (Plastic2oil 2019). Pyrolysis is used worldwide for as a waste-to-fuel thermal treatment technology, including the Sapporo Plastic Recycling establishing a fully commercial plastic liquefaction facility on the island of Hokkaido in 2000 with the scale to recycle 50 tonnes of mixed plastic waste a day (Klean Industries 2019). Other notable companies utilising pyrolysis for the waste-to-fuel process are Anhui Orsun Environmental Technologies, Blest, Dynamotive and Niutech Energy Ltd (Haig et al. 2017).

In the United States more than 137 million tons of MSW were landfilled back in 2015, out of which 26.01 million tons was plastic waste (US EPA 2019). Pyrolysis has the potential to decrease the use of landfills as an MSW management technique by 19% and decrease the consumption of conventional fuels. According to the figures reported by Plastic Energy, each tonne of end-of-life plastic PSW processed, 850 litres of chemical feedstock (pyrolysis oil) TACOIL is produced (Plastic Energy 2019). According to report by 4R Sustainability, Inc. (2011) one ton of MSW produces 264 gallons of consumer-ready fuel (around 1000 litres of pyrolysis oil). The average consumption of petroleum is 20.5 million barrels per day in the United States (Eia.gov 2019). Converting landfilled plastics into pyrolytic oil could reduce the petroleum consumption by 1.8% as well as reduce the air and water contamination. The GHG emissions associated with the use of waste plastics as a feedstock depend on the use from which that plastic is diverted. The bio-oil production from biomass pyrolysis may have other environmental impacts, for example increasing greenhouse gas (Bringezu et al. 2009). Products from PSW pyrolysis are also unpredictable at times and depend of the feedstock type. Hence, life cycle assessments must be conducted to identify the overall environmental impact of pyrolysis (Wang et al. 2015). The use of municipal solid waste (MSW) for oil and energy production instead of landfilling has a positive effect on the greenhouse gas emissions (GHG) equilibrium. Wang et al. (2015) examined the GHG emissions for both options—pyrolysis and landfilling of the MSW—resulting to 79% reduction of the GHG emissions when MSW were transferred to a fast pyrolysis plant for treatment. In addition, Gunamantha (2012) investigated the environmental impact for different scenarios of MSW treatments showing that incineration resulted in reducing the GHG from 374 kg CO_2-eq_/tMSW for landfilling to 61 kg CO_2-eq_/tMSW for incineration while energy recovery was estimated at 291 kWh in the latter case. Stepanov et al. (2018) examined the reduction of the environmental burden when MSW were sent for incineration instead of landfilling, showing that the GHG were reduced from 2.69 × 10^7^ kg CO_2-eq_/year to 1.22 × 10^5^ kg CO_2-eq_/year. On the other hand, the study of Demetrious and Crossin (2019) regarding the treatment of mixed plastics showed that the GHG via gasification-pyrolysis were 1.87 kg CO_2-eq_/kg processed, in comparison to the 0.0151 kg CO_2-eq_/kg processed emissions via landfilling, due to the large impact of the direct emission and the electricity. Similarly, the study of Zaman (2013) concluded that the global warming potential of pyrolysis-gasification process of the MSW was 1000.153 kg CO_2-eq_/tMSW compared with the 40.04723 kg CO_2-eq_/tMSW in case of landfill, despite the benefits for energy production via pyrolysis of the MSW. These results could be related to the GHG impact of the harmful residue of the pyrolytic process leading to productions of carbon dioxide and carbon monoxide during decomposition of final residue.

## LCA Standard Methodology, Description and Limitations

One of the techniques developed to assess and evaluate the possible environmental impacts of products and processes is LCA. It is an internationally standardized method that has been developed from chemical engineering principles and energy analysis (Hertwich et al. 2002). The International Standard of ISO 14040 (1997; 2006) regulates the practice and describes the principles, methodology and framework for conducting LCA and assists in identifying the parameters to improve the environmental aspects of products at various points in their life cycle. The analysis considers any option that influences the environment by consuming resources and releasing emissions which consequently generate waste streams. Generally, the impacts that are considered include resource use, human health and ecological impacts. LCA is an effective decision-making technique for waste management and treatment processes (Rigamonti et al. 2009). ISO 14040 (2006) defines the four basics for conducting an LCA study thus;

1. Goal and scope definition; where the objectives are defined and the extent of the study and the functional unit (FU) are set within the boundaries of the system.
2. Life cycle inventory (LCI) or Inventory Analysis: In this stage, mass and energy balances are developed and the inputs/outputs of the system are defined.
3. Life cycle impact assessment (LCIA): The impact and burdens are evaluated in this stage with a set magnitude and value with the aid of impact indicators.
4. Life cycle interpretation: This is the final stage where the study is systematically evaluated and conclusions with respect to scope and FU are derived.

**Fig. 1:**
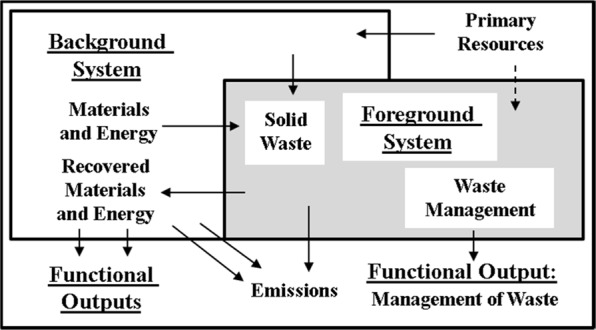
Foreground and Background systems used explicitly by the EA (UK). Source: Clift et al. (2000)

The LCA system boundaries establishes the processes included within the supply chain of fuel or products. The system boundaries must account for time, space and the functional unit (FU) chosen as a basis of comparison (Eriksson et al. 2002). It is paramount to distinguish between the ‘foreground’ system and the ‘background’ system. The former being a set of processes whose selection or mode of operation is affected directly by decisions based on the study (in this case waste management activities), whilst the latter is defined as all other processes that interact with the foreground system, usually by supplying or receiving materials and energy (Fig. 1).

LCA is conducted by establishing an inventory of inputs and outputs of the production system, assessing their potential environmental consequences, and interpreting the results in relation to the objectives of the assessment. However, the system boundary, initial assumptions and the FU chosen may affect the results interpretation and render comparison between LCA studies impossible. Results of global and regional LCA studies differ and might not appropriately represent the local conditions. Thus, comparing LCA studies is only possible if the assumptions and context of each study are the same. LCA has some limitations and is not a universal assessment technique. Typically, LCA does not account for the economic or social aspects of a product. Nevertheless, the international standards organisation (ISO) has released further guidelines over the LCA methodology by introducing the 14070 Standard series, such as the ISO 14071 (2014) and ISO 14072 (2014). The new guidelines account for additional requirements over the previous ISO 14066 (2011) as far as organization are concerned in reporting LCA results. Economical or socio-economical categories are now encouraging and assigned to numerical values in such cases. Factors such as visual pollution, odours, noise, destruction of the natural habitat, etc., are likely to be excluded from an LCA analysis, although these factors are important and must be considered in the decision-making process (Arena et al. 2003).

LCA methodology has been used for a variety of different systems and processes as a decision-making tool. LCA can be applied various assessment approaches, regarding the studied system and the system boundaries considered. Well-To-Wheel (WTW), Well-To-Pump (WTP) or Well-To-Tank (WTT) methodologies are used by the energy and fuel production sector to describe and assess the environmental impact of fuels, taking into account the use of product (that is WTW) or only the upstream process up to fuel storage before use (that is WTP). Collet et al. (2013) reviewed the environmental impact of biodiesel synthesis from microalgae considering both WTW and WTP analyses. Their analysis relied on the GHG and energy balance of the studied systems, excluding the social and economic aspects. However, they insisted on the importance of a common functional unit and system boundaries to allow comparison among the studied systems for assessments of similar scope and aim. The Joint Research Centre of EU released a technical report on the WTT and WTP pathways of different petroleum-derived fuels and biofuels, based on the ISO 14040 series Standards, establishing common LCA pathway analyses and comparison methodologies for the EU region. Cabeza et al. (2014) depicted LCA case studies for the building industry, and they implemented an extension by considering direct and indirect energy demands and cost analysis. Regarding the construction industry, fundamental LCA methodology focuses on the cradle-to-grave analysis and end-of-life recycling of the construction material, assessing the environmental impact of the construction. Through Life Cycle Energy Analysis (LCEA), energy demands were also included in the environmental and sustainability study. The study of Laurent et al. (2014a, 2014b) showed that LCA studies are dependent on the location and the local regulations, hindering the comparison between different life cycle assessments. The most common LCA analyses concerns cradle-to-gate and cradle-to-grave systems. These studies include the process from material extraction to disposal or recycling, respectively.

Madival et al. (2009) focused on a cradle-to-cradle LCA comparison between poly(lactic acid) (PLA), poly(ethylene terephthalate) (PET) and poly(styrene) (PS) as packaging materials, in which the analysis concerned the process from material extraction until disposal, and use for energy production or material replacement by recycling. In their study they focused on the GWP and the eco-toxicity burdens, as well as land occupation, showing that transportation stages of the materials had the major environmental impact and thus should be considered in the system boundaries of the LCA studies. Blengini et al. (2012) studied the credibility and acceptability of the LCA results, that are influenced by methodological assumptions and the local socio-economic constraints. The average consumption of petroleum is 20.5 million barrels per day in the United States (Converting landfilled plastics into pyrolytic oil could reduce the petroleum consumption by 1.8%.

## LCA Studies in Context of Plastic Solid Waste Management

Different software (computerized-aided solutions) systems are used to design and conduct the LCA studies for the pyrolysis process used for the plastic solid waste managements. The most commonly used for the research projects and technical assessments are reported in Table 2. Table 3 shows the avoided burdens via different waste management treatments of MSW, while Table 4 summarises major findings of some of the main published results of LCA studies encompassing PSW as part of the studied material flow. According to the published findings, thermochemical treatment could result to a sustainable solution for plastic solid waste management, due to the low values of all environmental burdens for all chosen FU.

**Table 2:** Commonly used LCA software packages

Software Package Name	Producing Company	
LCA	PER	The software assess all input flows to and from nature in a cradle to grave fashion. It is used commonly in combination to life cycle analysis.
SimaPro	PER	Users can collect, analyse and monitor the environmental performance of processes. The user can model LCA in a systematic way following the ISO 14040 recommendations.
Umberto	German ifu Hamburg.	This software visualizes material and energy flows. It can model complex structure with its graphic interface. It can also model production facilities in a company, processes and value chains.
Software Development Life Cycle (SDLC)	Stylus Systems Inc.	This package is also known as a linear sequential model, where activities such as system/information engineering are modelled.
Gabi	PE Europe	This software provides solutions for different problems regarding cost, environment, social and technical criteria, optimization of processes and manages external representation in these fields.

Adapted from Garcia-Serna et al. (2007)

**Table 3:** Avoided burdens considered for different case studies of waste management treatments of MSW

Source	Waste management technique	Avoided burdens
Eriksson et al. (2005)	Incineration Incineration with biological treatment Incineration with material recycling Landfilling	Consumption of primary energy production
Eriksson and Finnvedenb (2009)	Incineration	Use of fossil fuels for electricity and heat from a CHP unit
Bovea et al. (2010)	Recycling Biological treatment Landfilling	Use of virgin materials
		Use of fertilisers and electrical energy
		Electrical energy
Fruergaard and Astrup (2011)	Co-combustion of solid recovered fuels Anaerobic digestion Incineration	Use of fossil fuels for electricity and heat from a CHP unit
		Use of fossil fuels for electricity, heat and transportation and fertilisers by the digestate fraction.
		Use of fossil fuels for electricity and heat from a CHP unit
Iribarren et al. (2012)	Sequential pyrolysis and catalytic reforming (SPCR) Incineration Landfilling	Refinery gas, gasoline, diesel
Al-Salem et al. (2014a)	Incineration and materials recovery facility (MRF) Low temperature pyrolysis Vea Combi-Cracking hydrogenation reactor	Production of steam from natural gas and electricity from the grid. Production of virgin plastics, glass and steel from the MRF
		Petrochemical-based commercial products and production of steam from natural gas
		Commercial products from produced chemicals
Wang et al. (2015)	Pyrolysis	Coal, natural gas, diesel, gasoline

**Table 4:** Functional unit, global warming potential (GWP), acidification potential (AP), eutrophication potential (EP), human toxicity (HT) and photochemical ozone creation potential results of literature studies using advanced thermochemical processes

Reference	Thermo-chemical processes used	FU	GWP (kg CO_2-eq_)	AP (kg SO_2-eq_)	EP (kg PO_4_^3-^-_eq_)	HT (kg 1.4 DCB_eq_)	POCP (kg C_2_H_4_)
Rigamonti et al. (2009)	Incineration	1 ton MSW	−255 ~ −178	−2.4 ~ −2.3	–	−245 ~ −162	−0.21 ~ −0.17
Iribarren et al. (2012)	Pyrolysis	Production of 1 kg of gasoline blendstock	2.44	9.53	1.34	–	0.41
Gunamantha (2012)	Combination of gasification, anaerobic digestion and incineration	1 ton of solid waste treated	−168 ~ 188	−2.7968 ~ 0.0428	−0.1618 ~ 0.005	–	−0.1585 ~ 0.3898
Al-Salem et al. (2014a)	Incineration	137,303 tonnes per annum (tpa) MSW	1.85 ~ 0.25	5.75E−03 ~ 0.12 E−03	1.49E−03 ~ 0.2E−04	–	4.76E−04 ~ 0.23 E-04
Wang et al. (2015)	Pyrolysis	1 kg organic components in MSW	1194	1.6	0.1	–	–
Evangelisti et al. (2015)	Gasification and plasma gas cleaning, pyrolysis and combustion and gasification with syngas combustion	1 kg of municipal solid waste	0.465 ~ 0.698	−0.001 ~ −4.21E−04	6.94E−05 ~ 9.26E−04	−0.058 ~ 0.544	−8.44E−05 ~ −5.62E−05
Popiţa et al. (2017)	Incineration	197,000 Mg·y^−1^	−1.44E + 21	−9.63E + 16	6.30E + 14	−2.2E + 11	−7.50E + 16
Zhou et al. (2018)	Incineration	1 ton MSW	271	−0.748	–	–	−0.053
Demetrious et al. (2018)	Incineration	30,000 ton of material recovery facility residual waste	1.55 E + 07	1.01 E + 04	2.39 E + 03	–	–
Demetrious et al. (2018)	Gas-pyrolysis	30,000 ton of material recovery facility residual waste	2.30 E + 07	2.00 E + 04	4.79 E + 03	–	
Ardolino et al. (2018)	Gasification	1 kWh of recovered electricity	1.60–2.23	87E−06–120E−06	–	–	–
Stepanov et al. (2018)	Incineration	Waste from a geographical area in 1 year	1.13E + 08		-–	–	1.02E + 04
Chen et al. (2019)	Incineration	1 ton MSW	−0.648	−0.0037	−1.22E−05	–	−1.94E−04
Khoo (2019)	Pyrolysis	822,200 tonnes of treated plastic waste	625,500,000–748,500,00	96,000–109,000	–	–	–
Demetrious and Crossin (2019)	Gasification‑pyrolysis	1 kg of mixed plastic	1.87E + 00	2.43E−05	1.37E−04	–	2.93E−07

Song and Hyun (1999) conducted LCA study on the various recycle routes of PET bottles. Mathematical models for the waste (including PSW) recycling systems have been developed using the energy and material balances on each operation involved. The Jacobian matrix of partial derivatives representing the sensitivity of each environmental burden was used for an analysis. Khoo (2009) evaluated eight waste treatment technologies in Singapore. The impacts analysed were GWP, AP, terrestrial eutrophication and ozone photochemical formation. The greatest impacts were caused by the thermal cracking gasification of granulated MSW and the gasification of refuse-derived fuel (RDF), while the least were from the steam gasification of wood and the pyrolysis–gasification of MSW. The most cost-effective technique was identified to be the circulating fluidized bed (CFB) gasification of organic waste and the combined pyrolysis, gasification and oxidation of MSW.

Rigamonti et al. (2009) have analysed the material and energy recovery within MSW management systems to evaluate the most efficient and environmental results. Simapro 7 software, developed by PRè Consultants was used for the evaluation. Two characterisation methods were used; the cumulative energy demand (CED) and CML 2. CED investigates the energy demand of the process to estimate the total energy demand. Negative estimations are typically more favourable as they indicate the system studied is in credit (Al-Fadhlee and Al-Salem 2015). CML 2 is an LCA method developed by the CML (Centrum voor Milieuwetenschappen - Centre of Environmental Sciences, an institute of the Faculty of Science of Leiden University), it evaluates the environmental impacts through the process’s life. Several environmental impacts were considered such as; global warming potential (GWP), human toxicity potential (HTP), acidification potential (AP) (emissions of NOx, SOx and ammonia) and photochemical ozone creation potential (POCP). Three MSW integrated management systems were analysed, differing in the quantities of waste sent to material recovery and to energy recovery routes. The source separated collection scenarios were taken as 35, 50 and 60%. The results obtained showed that the optimum source-separated collection is 60% as the materials are recovered with high efficiency.

Iribarren et al. (2012) used LCA to evaluate the performance of the sequential pyrolysis and catalytic reforming (SPCR) of PE wastes. The objectives of the study were to assess environmental and energy characterization of the system, identify the processes with the highest contributions to the potential impacts, and compare the performance of the SPCR system with conventional waste management techniques such as landfilling and incineration. Seven impact potentials were considered for evaluation; CED, abiotic depletion (ADP), AP, eutrophication (EP), GWP, ozone layer depletion (ODP), and photochemical oxidant formation (POFP). The result showed that the traditional hierarchical approach is accurate as the recycling and recovery were identified as better options compared with conventional plastic waste treatments; landfilling and incineration. The SPCR products showed lower impacts in all categories except GWP (for gasoline and diesel) compared with products from the conventional techniques. Minimising the direct emissions would improve the GWP.

Gunamantha (2012) analysed five municipal solid waste treatment scenarios; landfilling system with energy recovery, a combination of incineration and anaerobic digestion, combined gasification and anaerobic digestion, direct incineration, direct gasification. These scenarios were compared with the existing landfilling system. In the study, gas emissions such as CO_2_, CO, CH_4_, N_2_O, NO_2_, NH_3_, SO_2_, H_2_S, HF, HCl, and NMVOC were selected as the objects for assessment and were allocated into impact categories; GWP, AP, eutrophication, and photochemical oxidant formation. In terms of global warming, eutrophication and photochemical oxidant production direct gasification was identified to be the most feasible with savings of 168 kg CO_2_ eq/FU, 0.17 kg PO_4_ eq/FU, and 0.16 kg ethylene eq/FU, respectively. While in terms of acidification, gasification and anaerobic digestion gave the highest value of saving 2.8 kg SO_2_ eq/FU.

Al-Salem et al. (2014a) evaluated the waste management system in the Greater London area using the GaBi software. Waste produced in Greater London was sent to a dry materials recovery facility and to an incineration unit with combined heat and power production. This waste treatment technique was compared with a landfill scenario and the study showed that the actual waste management system in Greater London has a lower environmental impact than the landfilling. The paper also analysed two alternative technologies; pyrolysis and hydrogenation. The use of hydrogenation resulted in the highest savings in terms of eutrophication potential due to avoided naphtha production. In a follow-up study and implementing the same methodology, PO PSW was used as a feedstock to a pyrolysis process for the State of Kuwait in Al-Salem (2014b). The waste feedstock used has reduced both the GWP and AP by over 30% for the whole country when compared with the baseline scenario and in a combination to incineration for energy recovery. The LCA also confirmed that sustainable management can be achieved for the studied systems since products can replace those of the largest refinery in the country in an integrated manner.

Later, Wang et al. (2015) have investigated the environmental impacts of an MSW pyrolysis plant in North Carolina (USA). LCA was conducted to assess the environmental impacts of production, upgrading and usage of bio-oil from MSW using GaBi software. The impacts of pyrolysis were compared with anaerobic digestion, incineration and landfilling for MSW. Pyrolysis for bio-oil was identified to have the least impact, while the landfilling for treating the MSW causes the most undesirable impact on the environment. Evangelisti et al. (2015) compared the environmental impacts of three dual-stage technologies; gasification and plasma gas cleaning, pyrolysis and combustion and gasification with syngas combustion. These techniques were compared with conventional MSW treatments which were landfilling with electricity production and incineration with electricity production. Results show that the two-stage gasification and plasma process has better environmental performance than the conventional techniques and modern incineration plant, which was demonstrated by a plant in Lincolnshire (UK). The advantage of gasification with plasma process is mainly from the higher net electrical efficiency. It should be noted that the gasification gas combustor process has a GWP of 0.18 kg CO_2_ eq/kWh (electrical production). This accounts for only 30% of the Sheffield incineration plant and 75% of the North Hykeham incineration plant. The result of the study showed that the two-stage gasification and plasma process is more environmental solution for the MSW treatment compared with incineration processes, for all the impact categories taken into the account.

Chen et al. (2019) conducted the environmental, energy and economic analysis of integrated treatment of MSW and sewage sludge (SS) in China. Four scenarios were studied including mono-incineration of MSW (Case 1) and SS (Case 2), co-incineration of SS and MSW by traditional (Case 3) and integrated ways (Case 4), by means of LCA, CED and TEA method. It was found that Case 4 had the most optimistic effect on climate change and resources (−1.44 kg CO_2-__eq_ and −18 MJ, respectively) corresponding to the end-point categories. From an energy perspective, Case 4 demonstrated the most desirable performance of energy efficiency, and significantly saves non-renewable energy (0.21 t coal per ton feedstock compared with Case 3). From an economics point of view, Case 4 is preferred with the best profit, reducing 79.08% of cost in coal than that in Case 3. It was concluded that these results provide an understanding of developing an effective approach for co-treating MSW and SS.

Ardolino et al. (2018) conducted a study providing a Life Cycle Inventory model for the fluidised bed gasification of wastes. All the data had been obtained from a pilot scale fluidized bed gasifier, fed with ten types of waste and biomass, under a wide range of operating conditions. The Crucial relationships between process- and waste-specific parameters were well-defined. The model quantifies the key inputs and outputs of the gasification process (emissions, energy recovery, ash disposal, resource consumptions), generating high-quality data that could contribute to advance life cycle assessment modelling of waste gasification. Lastly, some case studies were implemented in the EASETECH software to illustrate the model applicability, assess the role of main parameters, and compare the environmental performances of gasification power units with that of the European electricity mix. The performances seem to be largely affected by the metal contents in the waste-derived fuels, whilst the model results to a restricted extent are sensitive to the equivalence ratio and the net electrical efficiency of the energy conversion.

Demetrious et al. (2018) compared the alternative methods for managing the residual of material recovery facilities using LCA in Sydney, Australia. In this study, the environmental performance of the material recovery facilities’ residual waste was evaluated using an LCA that approximates the potential impacts of acidification, climate change, eutrophication and photochemical oxidation. A sensitivity analysis established different waste fractions of material recovery facilities (MRF) residual waste composition. The results showed that landfill had the lowest GHG emissions irrespective of whether credits offset electricity, and of the carbon accounting methods used to measure biogenic carbon dioxide. It was also found that landfill had the lowest acidifying emissions, however, the waste-to-energy technologies performed better in diminishing eutrophying and photochemical oxidation emissions. Aggregated by normalization and weightings, landfilling was observed to have the lowest single score. The study conveyed electricity generation potentials through thermal turbine, synthetic gas engine and landfill gas combustion, and concluded incineration to have highest electricity generation potential, followed by gasification-pyrolysis.

## Major Findings, and Way Forward, Detailing Research Gaps in Area

Astrup et al. (2015) reviewed 136 journal articles regarding the LCA of the waste to energy technologies such as; incineration, co-combustion, pyrolysis and gasification. They have analysed existing LCA studies to identify the most important methodological aspects and technology parameters, and to provide recommendations for the LCA assessments. Most of the case studies analysed incineration and only a few addressed pyrolysis. Not all papers provided detailed description of goal and scope of the assessment, the technologies included, and the calculation principles applied. Furthermore, in very few studies the reported results could be verified that limits the application of the inventory data and results. LCA guidelines outline the main assessment principles, but little methodological consistencies exist between LCA studies in literature. Results of the LCA studies based on similar waste type and technology vary considerably.

Some LCA studies suggest that the anaerobic digestion is preferable (e.g. Khoo 2009), while others favour waste incineration (e.g. Manfredi et al. 2011; Fruergaard and Astrup 2011). Therefore, the given guidelines still allow the room for interpretation (Laurent et al. 2014a, 2014b). Technology modelling principles, LCA principles, impact assessment methodologies and emission levels vary significantly between LCA studies (Laurent et al. 2014a). The detailed waste composition and type used in the study is important for the framework of the assessment. In the review by Astrup et al. (2015) only 70% of the case-studies provided a detailed description of the material fractions present in the waste, while only 44% provided information about the chemical composition of the waste. The lack of detailed descriptions in the studies limits the LCA modelling as emissions are affected by the waste input composition. Few of the LCA studies provide enough description of the LCA modelling scope and of the technologies included in the assessment. Omitting the information limits the linking between the functional unit, the waste composition and the waste to energy technology assessed. Also, the key parameters such as air-pollution-control, residue management, and capital goods were omitted in many published past works. In the papers where the description of LCA modelling approaches is weak, the calculations cannot be reproduced or assessed for validity. This significantly limits the application of the LCA results for decision makers and limits the value of LCA studies for the implementation of waste to energy technologies in society. In order to evaluate the validity of the LCA conclusions, the studies should assess parameter and scenario uncertainties. Despite this, 46% of the case-studies do not include uncertainty assessments. Only 29% of the studies included sensitivity analysis on selected parameters, while scenario uncertainties were only evaluated in 41% cases (Astrup et al. 2015).

There have been various LCA studies conducted under protection and non-disclosure agreements that prohibit the public from knowing the end results. These include various major projects around the world that are concerned with commercial and urban development. Social and economic impacts are two main categories that need to be addressed in future studies concerning PSW management. Furthermore, one major impact that needs to be implemented in future studies is geographical location. Various processes and systems depend on the geographical location of a country or a production line, etc. This aspect, in combinations with the impact of various renewable energy resources that depend on the geographical location of many societies, can be added to the assessment categories in the near future.

## Conclusions and Recommendations

Plastic solid waste remains one of the major concerns globally due to the environmental impact, as it can lead to long-term soil and groundwater pollution. Pre-treatment and recycling have been proven beneficial for reducing their impact, however, the increasing amounts of plastic waste and the low percentage used as recyclable plastic highlight the importance of post-treatment of the PSW. LCA is a developed tool for assessing the weight of environmental pollution and analysing the avoided burdens based on the processes taking place on waste management. The steadily increasing inventory (LCI) allows detailed analysis on the allocation of burdens and pollutants on each step of the waste management process, and the selection of the appropriate sustainable method. LCA studies are usually augmented via the sensitivity analysis studies for more detailed results on the behaviour of the concerned systems and the selection of the optimal process conditions or decreasing system uncertainty. Published studies on the environmental impact of PSW have shown that thermochemical post-treatments, such as gasification, incineration or pyrolysis, result to further decrease of the environmental effects, in comparison to landfilling. Furthermore, pyrolysis offers the advantage of bio-oil and char production of high calorific value, which can be used as fuels either for internal consumption of the plant or in other systems as substitution to fossil fuels. Hence, pyrolysis agrees with the environmental guidelines drawn by the ISO 14040 and 14044 standards to promote sustainable environmental solution on waste management. However, LCA is not univocally addressed for the environmental assessment, but is rather considered an integrated tool accounting for the life cycle cost of the proposed methodology and the overall process. Lack of market values on emissions and pyrolysis fuel products result to debatable results, which are subject to considered system boundaries, assumptions and functional unit. This review introduces pyrolysis as a studied and robust methodology for PSW post-treatment for minimization of the environmental burdens of the process, and emphasizes the importance of drawing a systematic scheme of LCA analysis on PSW management. Pyrolysis is an advanced waste treatment technique and this review has potentially a key role to play on the development of a strategic planning in which all advantages of pyrolysis will be considered.

## Abbreviations

ADP: Abiotic Depletion Potential
AP: Acidification Potential
C-C: Carbon to Carbon
CED: Cumulative Energy Demand
CFB: Circulating Fluidized Bed
CH4: Methane
CHP: Combined Heat and Power
CO2: Carbon Dioxide
CO: Carbon Monoxide
CV: Calorific Value
EIA: Environmental Impact Assessment
EP: Eutrophication Potential
EU: European Union
FU: Functional Unit
GHG: Greenhouse Gas
GWP: Global Warming Potential
HCl: Hydrogen Chloride
HF: Hydrogen Fluoride
HHV: Higher Heating Value
H2S: Hydrogen sulphide
HTP: Human toxicity potential
ISO: International Standards Organisation
LCA: Life Cycle Assessment
LCEA: Life Cycle Energy Analysis
LCI: Life Cycle Inventory
LCIA: Life Cycle Impact Assessment
MPW: Municipal Plastic Waste
MSW: Municipal Solid Waste
MRF: Material Recovery Facilities
NMVOC: Non-Methane Volatile Organic Compound
NH3: Ammonia
NOx: Nitrogen Oxides
N2O: Nitrous Oxide
ODP: Ozone Depletion Potential
PAHs: Polycyclic Aromatic Hydrocarbons
PCDD: Polychlorinated Dibenzo Para Dioxins
PCDF: Polychlorinated Dibenzo Furans
PE: Polyethylene
PET: Polyethylene Terephthalate
PLA: Polylactic Acid
PMMA: Polymethylmetacrylate
PO: Polyolefin
POCP: Photochemical Ozone Creation Potential
POFP: Photochemical Oxidant Formation Potential
PP: Polypropylene
PS: Polystyrene
PSW: Plastic Solid Waste
PU: Polyurethanes
PVC: Polyvinyl Alcohol
RDF: Refuse-Derived Fuel
SDLC: Software Development Life Cycle
SOx: Sulphur Oxides
SPCR: Sequential Pyrolysis and Catalytic Reforming
SS: Sewage Sludge
SW: Solid Waste
TCT: Thermo-Chemical Treatment
TEA: Techno-Economic Assessment
VOCs: Volatile Organic Compounds
WTP: Well-To-Pump
WTT: Well-To-Tank
